# Towards universal systems for recombinant gene expression

**DOI:** 10.1186/1475-2859-9-27

**Published:** 2010-04-30

**Authors:** Hans Peter Sørensen

**Affiliations:** 1 Danish-Chinese Centre for Proteases and Cancer, Danish National Research Foundation, Aarhus University, Department of Molecular Biology, Gustav Wieds Vej 10C, DK 8000 Aarhus, Denmark

## Abstract

Recombinant gene expression is among the most important techniques used both in molecular and medical research and in industrial settings. Today, two recombinant expression systems are particularly well represented in the literature reporting on recombinant expression of specific genes. According to searches in the PubMed citation database, during the last 15 years 80% of all recombinant genes reported on in the literature were expressed in either the enterobacterium *Escherichia coli *or the methylotropic yeast *Pichia pastoris*. Nevertheless, some eukaryotic proteins are misfolded or inadequately posttranslationally modified in these expression systems. This situation demands identification of other recombinant expression systems that enable the proper expression of the remaining eukaryotic genes. As of now, a single universal system allowing expression of all target genes is still a distant goal. In this light, thorough experimental screening for systems that can yield satisfying quantity and quality of target protein is required. In recent years, a number of new expression systems have been described and used for protein production. Two systems, namely *Drosophila melanogaster *S2 insect cells and human embryonic kidney 293 (HEK293) cells stably expressing the EBNA-1 gene, show exceptional promise. The time has come to identify a few well-performing systems that will allow us to express, purify, and characterize entire eukaryotic genomes.

## Introduction

**Figure 1:**
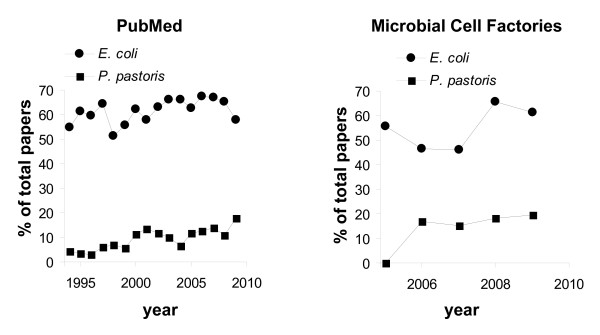
**Percentage of all proteins reported to be produced in *E. coli *or *P. pastori*s recombinant gene expression systems based on literature searches**. The PubMed citation database http://www.ncbi.nlm.nih.gov/pubmed/ or the journal *Microbial Cell Factories* http://http://www.microbialcellfactories.com/ was searched using "recombinant protein" only or in combination with "*Escherichia coli*" or "*Pichia pastoris*" as a keyword. Data represent the percent of total citations found by searching using "recombinant protein" that was found when that term was combined with one of the two organism names. Citation data are presented for the last 15 years for PubMed and the last 5 years for *Microbial Cell Factories*.

*Escherichia coli *was the first host to be used for recombinant gene expression almost 40 years ago [1]. Within the past 15 years, *Pichia pastoris *has successfully entered the scene and is now the second most-used host for recombinant gene expression. Based on searches of the PubMed citation database, the use of *P. pastoris *as an expression host has increased from 4% to 17% of the total recombinant genes reported on from 1995 to 2009 (Fig. 1). Within the same time period, the usage of *E. coli *as an expression host remained constant, with approximately 60% of the recombinant genes reported on in journals indexed in PubMed being expressed in *E. coli*. Similar trends prevailed when analyzing publications in the journal *Microbial Cell Factories *during the period from 2005 to 2009 (Fig. 1). Several other expression systems are widely used, but to a lesser extent than *E. coli *and *P. pastoris*. So, why are *E. coli *and *P. pastoris *particularly suited for recombinant gene expression?

Protein produced from a recombinant gene in *E. coli *as a soluble and functional product with high yield is the ideal situation for most research and industrial protein production purposes [2-4]. However, this situation is far from realistic for all recombinant gene products. In particular, proteins derived from eukaryotes are prone to inclusion body formation and low yields. This outcome can be explained by the fact that the rate of gene translation in *E. coli *is 4- to 10-fold higher than in eukaryotes [5]. Correct formation of disulfide bonds, protein folding, and protein function must be carefully assessed both when the proteins have been recombinantly produced in *E. coli *in a soluble form, when they have been refolded *in vitro*, or obtained from any other source [6]. Further, *E. coli *should not be used as the expression system if posttranslational modifications (PTMs) are of importance for the study or purpose of the protein because these microbes are unable to incorporate PTMs, including N-linked glycan chains. However, when it is able to both properly form and posttranslationally modify a protein, *E. coli *is the perfect expression host, allowing fast and inexpensive preparation of heterologous proteins.

Many of the advantageous properties of *E. coli *are also offered by *P. pastoris*, a methylotropic yeast that can exploit methanol as its only carbon source [7,8]. As compared with *E. coli*, *P. pastoris *folds most eukaryotic proteins more efficiently and forms disulfide bonds correctly. Cultivation of *P. pastoris *in methanol-containing medium results in strong upregulation of the promoter of the alcohol oxidase I (AOX1) gene. This strong and tightly regulated promoter is therefore incorporated into the majority of vectors for expression of recombinant genes in *P. pastoris *[9]. When using *P. pastoris*, the recombinant gene product is typically engineered with a signal sequence to facilitate secretion, and the vectors used are selected to integrate into the genome [9]. However, the N-linked glycan chains added posttranslationally to proteins produced in *P. pastoris *are substantially different from the modifications added by mammalian cells [10].

## Discussion

Given that we have these two excellent systems that together over the last 15 years have been used to express up to 80% of all reported recombinant genes, what else is still needed? Imagine setting up a laboratory working to express recombinant eukaryotic genes for biochemical and structural analysis. Which recombinant expression systems need to be implemented? Numerous important factors must be considered before expression of a recombinant gene is attempted. What is the mass of the polypeptide? Multi domain proteins are typically more difficult to produce than the alternative single domain deletion mutants. Does the protein contain any disulfide bonds? Proteins with disulfide bonds would most likely not be correctly folded in *E. coli *[11]. Are any PTMs required for protein folding, stability, or function, and what is the final destination of the protein--secreted, in the cytoplasm, or incorporated into the membrane?

Based on published studies, *E. coli *and *P. pastoris *enable the expression of most recombinant genes. However, one or two alternative methods would be required to fill the final gap and enable expression of genes that could not be expressed in either *E. coli *or *P. pastoris*. Here, fast and relatively inexpensive expression systems are required. What properties that are unsatisfied by *E. coli *and *P. pastoris *are required by these systems? First, alternative expression systems should be able to correctly fold the proteins that require incorporation of PTMs, including glycan chains. Second, an alternative system should simply enable protein production at a decent yield when *E. coli *and *P. pastoris *expression systems fail. The choice of expression system is also highly dependent on the type of target protein (soluble, membrane bound, multi domain, containing disulfide bonds, etc.), as well as the intended use of the product. Proteins produced by recombinant methods are typically used in structural studies, *in vitro *activity assays, as antigens for antibodies, for *in vivo *studies, and as drugs or as targets for generating drugs. These different applications have different requirements for both quantity and quality.

Several important expression systems in addition to *E. coli *and *P. pastoris *have been described. The most prominent are insect cell expression systems, yeasts other than *P. pastoris*, mammalian cell lines, and a few prokaryotes other than *E. coli *(e.g., *Bacillus subtilis*) [12]. Are any of these expression systems significantly more successful than *E. coli *or *P. pastoris*, thereby representing an alternative to those two systems? Focusing on recombinant genes of eukaryotic origin, two systems deserve special attention. First, the insect cell line *Drosophila melanogaster *Schneider 2 (S2) has been increasingly utilized over the past few years for the production of heterologous proteins. This system takes advantage of stable integration of up to 1,000 target gene copies following transient transfection and antibiotic selection procedures. *D. melanogaster *S2 cells are cultivated at 28°C without any special requirements and yield appropriately processed and biologically active proteins [13,14]. Second, transiently transfected mammalian cell lines cultivated in suspension cultures under serum-free conditions are a promising candidate. Large-scale transfection of mammalian cells has gained increasing interest because of it-can quickly and inexpensively produce proteins [15]. In fact, such processes allows for milligram to gram amounts of protein to be produced in a short period of time (2-3 weeks). Several reports support the use of polyethylenimines for the transfection of human embryonic kidney 293 cells stably expressing the EBNA-1 gene (e.g., HEK293 6E) [15,16]. These cells are potentially suited for use in high-throughput recombinant gene expression facilities because of the fast and easy transient transfection procedure [17].

The use of screening of several expression systems would significantly improve the hit-rate for obtaining correctly folded, biologically active protein in high yield. High-throughput screening facilities for recombinant gene expression should at a minimum assess the expression of a target gene in *E. coli *and 2-3 eukaryotic expression systems. A laboratory with the ability to test expression of recombinant genes in *E. coli*, *P. pastoris*, *Drosophila *S2, and HEK293 EBNA systems would be perfectly equipped to successfully express most recombinant target genes.

Why do we need several hosts for recombinant gene expression? In the perfect scenario, one universal expression system would enable expression of all possible recombinant genes in a fast, inexpensive, and proper manner with respect to yield, folding, and biological activity. However, because of the limitations of existing systems, we are still far from that goal. It is therefore necessary to carefully evaluate the properties of every new target and to experimentally screen the most promising expression hosts.

## Conclusions

Because 80% of all reported recombinant genes are expressed in either *E. coli *or *P. pastoris*, a modern research laboratory would be well equipped for recombinant gene expression by implementing these two systems. However, *E. coli *and *P. pastoris *are inadequate particularly for expression of many eukaryotic genes. Alternative systems useful for expression of eukaryotic genes have been described, but are less frequently used. Here I suggest the *D. melanogaster *S2 and HEK293 EBNA-1 expression systems as the most promising alternatives to *E. coli *or *P. pastoris*. We are still far from the "one-host-for-all-recombinant-gene-expression" era, and the most suitable system for a particular target protein should be determined empirically among qualified candidates.

## List of abbreviations

HEK293: human embryonic kidney 293; PTM: posttranslational modification; S2: *D. melanogaster *Schneider 2 insect cell line

## Competing interests

The authors declare that they have no competing interests.
